# Antitumor Effects and Related Mechanisms of Penicitrinine A, a Novel Alkaloid with a Unique Spiro Skeleton from the Marine Fungus *Penicillium citrinum*

**DOI:** 10.3390/md13084733

**Published:** 2015-07-31

**Authors:** Qin-Ying Liu, Tong Zhou, Yang-Yang Zhao, Li Chen, Mei-Wei Gong, Qi-Wen Xia, Min-Gang Ying, Qiu-Hong Zheng, Qi-Qing Zhang

**Affiliations:** 1 Institute of Biomedical and Pharmaceutical Technology, Fuzhou University, Fuzhou 350002, China; E-Mails: liuqinyingbio@163.com (Q.-Y.L.); vickychou90220@163.com (T.Z.); zhaoyangyang0929@163.com (Y.-Y.Z.); 742946642@qq.com (M.-W.G.); xiaqiwen0917@163.com (Q.-W.X.); 2 Fujian Provincial Key Laboratory of Tumor Biotherapy, Fujian Provincial Tumor Hospital, Fuzhou 350014, China; E-Mail: yingmg@163.com; 3 Institute of Biomedical Engineering, Chinese Academy of Medical Science, Peking Union Medical College, Tianjin 300192, China

**Keywords:** penicitrinine A, marine-derived fungus, human malignant melanoma cell A-375, anticancer activity, apoptosis, anti-metastatic

## Abstract

Penicitrinine A, a novel alkaloid with a unique spiro skeleton, was isolated from a marine-derived fungus *Penicillium citrinum*. In this study, the isolation, structure and biosynthetic pathway elucidation of the new compound were described. This new compound showed anti-proliferative activity on multiple tumor types. Among them, the human malignant melanoma cell A-375 was confirmed to be the most sensitive. Morphologic evaluation, apoptosis rate analysis, Western blot and real-time quantitative PCR (RT-qPCR) results showed penicitrinine A could significantly induce A-375 cell apoptosis by decreasing the expression of Bcl-2 and increasing the expression of Bax. Moreover, we investigated the anti-metastatic effects of penicitrinine A in A-375 cells by wound healing assay, trans-well assay, Western blot and RT-qPCR. The results showed penicitrinine A significantly suppressed metastatic activity of A-375 cells by regulating the expression of MMP-9 and its specific inhibitor TIMP-1. These findings suggested that penicitrinine A might serve as a potential antitumor agent, which could inhibit the proliferation and metastasis of tumor cells.

## 1. Introduction

Malignant melanoma is one of the few cancers that have displayed an increasing incidence and, more importantly, a steady mortality rate over the past decade [1,2]. Even if surgery represents the cure in the early phase of disease, the prognosis in the metastatic phase remains very poor. Recently, immune checkpoint blockade with monoclonal antibodies directed at the inhibitory immune receptors CTLA-4 and PD-1 has emerged as a successful treatment approach for patients with advanced melanoma [3]. However, chemotherapy remains an essential treatment option [4]. Moreover, recurrence/metastasis is the major cause of death in patients with malignant melanoma cancer. Therefore, novel potent drugs, especially anti-metastasis drugs, would be urgently needed for the treatment of refractory or relapsing melanoma cancer patients [5].

Natural product compounds have substantial structural diversity and frequently afford new mechanisms of biological activity [6]. As a result, it is believed that a rich source of anticancer drug candidates could be obtained from natural products [5]. Almost all of the current natural anticancer drugs were either isolated or derived from plants and terrestrial microorganisms. However, after 50 years of intensive screening from plants and terrestrial microbes, the pace of natural products’ discovery and development with a unique scaffold has dramatically declined over the last two decades [7]. Recent trends in drug discovery emphasize that marine microorganisms are a potentially productive source of novel secondary metabolites and have great potential to increase the number of marine natural products in clinical trials [8]. The oceans are highly complex environments and house a diverse assemblage of microbes that occur in environments with extreme variations in pressure, salinity, and temperature [9]. Marine microorganisms have attracted great attention since they have developed unique metabolic and physiological capabilities that not only ensure survival in extreme habitats but also offer the potential to produce compounds with antitumor and other interesting pharmacological activities that would not be observed in terrestrial microorganisms [10,11,12]. Recently, several synthetic analogues with a promising anti-proliferative activity are reported [13,14,15,16,17,18].

In our study, more than 300 microbial strains isolated from sediment samples collected from the Min River estuary in China were screened for cytotoxicity against cancer cells. Among them, a fungal strain identified as *Penicillium citrinum* exhibited significant cytotoxic activity. We investigated the secondary metabolites of this fungus and obtained a novel alkaloid, penicitrinine A with a unique spiro skeleton. It is the first metabolite biosynthesized from citrinin and tetramic acid derivatives in nature. The chemical structure of this compound was determined according to 1D, 2D NMR and HRESIMS spectroscopic data. We investigated the cytotoxic effect of penicitrinine A on human melanoma A-375 cells and the detail mechanism in its regulation of apoptosis and metastasis. The results confirmed that penicitrinine A exhibited potent pro-apoptosis and anti-metastatic activity in human melanoma A-375 cell line, indicating that it might serve as a potential antitumor lead compound.

## 2. Results and Discussion

### 2.1. Structure Elucidation

**Figure 1:**
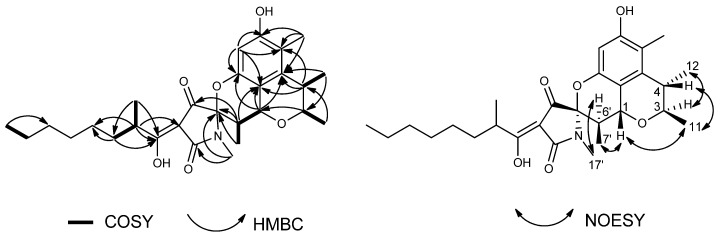
Key COSY, HMBC and NOESY correlations of penicitrinine A.

**Figure 2:**
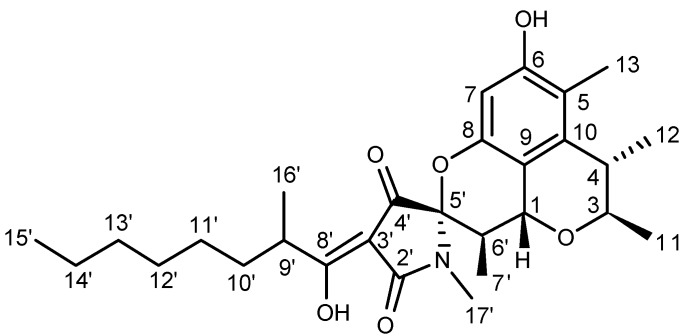
Chemical structure of penicitrinine A.

Penicitrinine A was obtained as yellow oil and analyzed to have the molecular formula C_28_H_39_NO_6_ through negative HRESIMS (*m*/*z*: 484.2711 [M − H]^−^, Calcd for C_28_H_38_NO_6_: 484.2705). Its NMR data (Table 1), combined with DEPT and HMQC spectrum analyses, revealed twenty-eight carbon signals, including seven methyls, five methylenes, six methines, and ten quaternary carbons. The 1D-NMR data of penicitrinine A mixed data of penicitrinol C [19] with that of penicillenol A_1_ [20], except for an obviously downfield shift of C-5′ (from 68.7 CH to 94.0 qC) [20], an obviously upfield shift of C-6′ (from 66.6 CH to 35.7 CH) [20], and the absence of an acetonyl group [19]. The COSY correlations (Figure 1) of H-6′ with H-1 and H-7′, and the HMBC correlations (Figure 2) from H-6′ to C-4′, C-5′, and C-9, and from H-7′ to C-5′ suggested that two monomers were finally connected through C-1 and C-6′ as well as 8-OH and C-5′. Furthermore, the relative configurations of penicitrinine A were revealed through the NOESY experiment. The NOESY correlations (Figure 2) of H-1 with H-7′ and H-11, H-11 with H-4, H-3 with H-12, and H-6′ with H-17′ indicating that H-1, H-4, 11-CH_3_ and 7′-CH_3_ were on the same side, whereas H-3, 12-CH_3_, H-6′ and N-CH_3_ were on the opposite side. Therefore, the structure of penicitrinine A was elucidated as shown (Figure 2).

**Table 1:** 500 MHz ^1^H and 125 MHz ^13^C-NMR data for penicitrinine A (in CDCl_3_).

Position	δ_H_ (*J* in Hz)	δ_C_
1	5.03 (1H, d, 11.0)	62.9 CH
3	4.14 (1H, q, 6.8)	75.1 CH
4	2.62 (1H, q, 6.8)	34.8 CH
5		115.3 qC
6		154.2 qC
7	6.08 (1H, s)	100.3 CH
8		149.2 qC
9		111.4 qC
10		136.9 qC
11	1.40 (3H, d, 6.8)	18.7 CH_3_
12	1.21 (3H, d, 6.8)	22.4 CH_3_
13	2.05 (3H, s)	10.1 CH_3_
2′		173.9 qC
3′		99.9 qC
4′		191.4 qC
5′		94.0 qC
6′	2.29 (1H, dq, 11.0, 6.8)	35.7 CH
7′	0.99 (3H, d, 6.8)	10.6 CH_3_
8′		192.3 qC
9′	3.59 (1H, m)	36.2 CH
10′	1.69 (1H, m)	34.0 CH_2_
	1.47 (1H, m)	
11′	1.24~1.37 (2H, m)	27.3 CH_2_
12′	1.24~1.37 (2H, m)	29.3 CH_2_
13′	1.24~1.37 (2H, m)	22.7 CH_2_
14′	1.24~1.37 (2H, m)	31.8 CH_2_
15′	0.87 (3H, t, 7.0)	14.2 CH_3_
16′	1.17 (3H, d, 6.9)	17.3 CH_3_
17′	2.90 (3H, s)	23.1 CH_3_

### 2.2. Biosynthesis of Penicitrinine A

**Figure 3:**
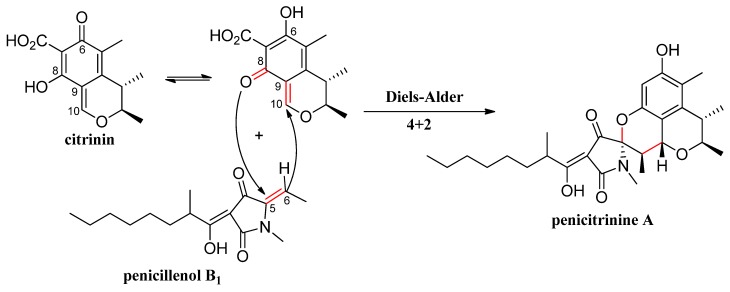
Plausible biosynthetic pathway of penicitrinine A.

Our previous chemical investigation of this strain reported the isolation of sixteen citrinin derivatives [19,21,22] and four tetramic acid derivatives [22], among which citrinin and penicillenol B_1_ seemed to be the biosynthetic precursors of penicitrinine A. During this biosynthesis process, the Diels-Alder reaction was involved in these two compounds. To clearly explain the biogenetic origin of penicitrinine A, a plausible biosynthetic pathway is proposed in Figure 3.

### 2.3. Penicitrinine A Inhibits the Proliferation of Multiple Tumor Types

**Figure 4:**
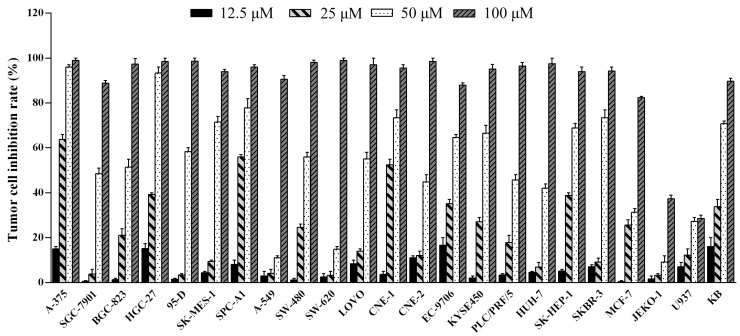
Tumor cell-growth inhibitory activity of penicitrinine A. Twenty-three tumor cell lines were treated with penicitrinine A for 48 h at concentrations from 12.5 to 100 μM. The data are presented as means ± SD from five independent experiments.

The cytotoxic effect of penicitrinine A was evaluated in a panel of twenty-three cancer cell lines derived from ten different types of tumors. As shown in Figure 4, different tumor cell lines had different levels of proliferation inhibition after treated with gradient concentrations of penicitrinine A for 48 h. The most sensitive cell lines were malignant melanoma cell line A-375, lung cancer cell line SPC-A1 and stomach cancer cell line HGC-27 with IC_50_ values of 20.12 μM, 28.67 μM and 29.49 μM, respectively (Table 2). Since the A-375 cell line was the most sensitive, we finally chose it as the target cell line for further study.

**Table 2:** Inhibition of tumor cell-growth activity of penicitrinine A.

Human Cancer Type	Cell Line	IC_50_ (μM)	Human Cancer Type	Cell Line	IC_50_ (μM)
Stomach cancer	SGC-7901	>50	Liver cancer	PLC/PRF/5	46.13
	BGC-823	44.58		Huh-7	49.87
	HGC-27	29.49		SK-HEP-1	33.20
Lung cancer	95-D	47.50	Nasopharynx cancer	CNE-1	31.76
	SK-MES-1	42.40		CNE-2	45.17
	SPC-A1	28.67	Esophagus cancer	EC-9706	36.14
	A-549	>50		KYSE450	38.89
Colon cancer	SW-480	41.59	Breast cancer	SKBR-3	41.88
	SW-620	>50		MCF-7	>50
	LOVO	42.33	Lymphoma cancer	Jeko-1	>50
Malignant melanoma	A-375	20.12		U937	>50
Oral epidermoid carcinoma	KB	35.25			

### 2.4. Penicitrinine A Inhibits the Proliferation of A-375 Malignant Melanoma Cells

**Figure 5:**
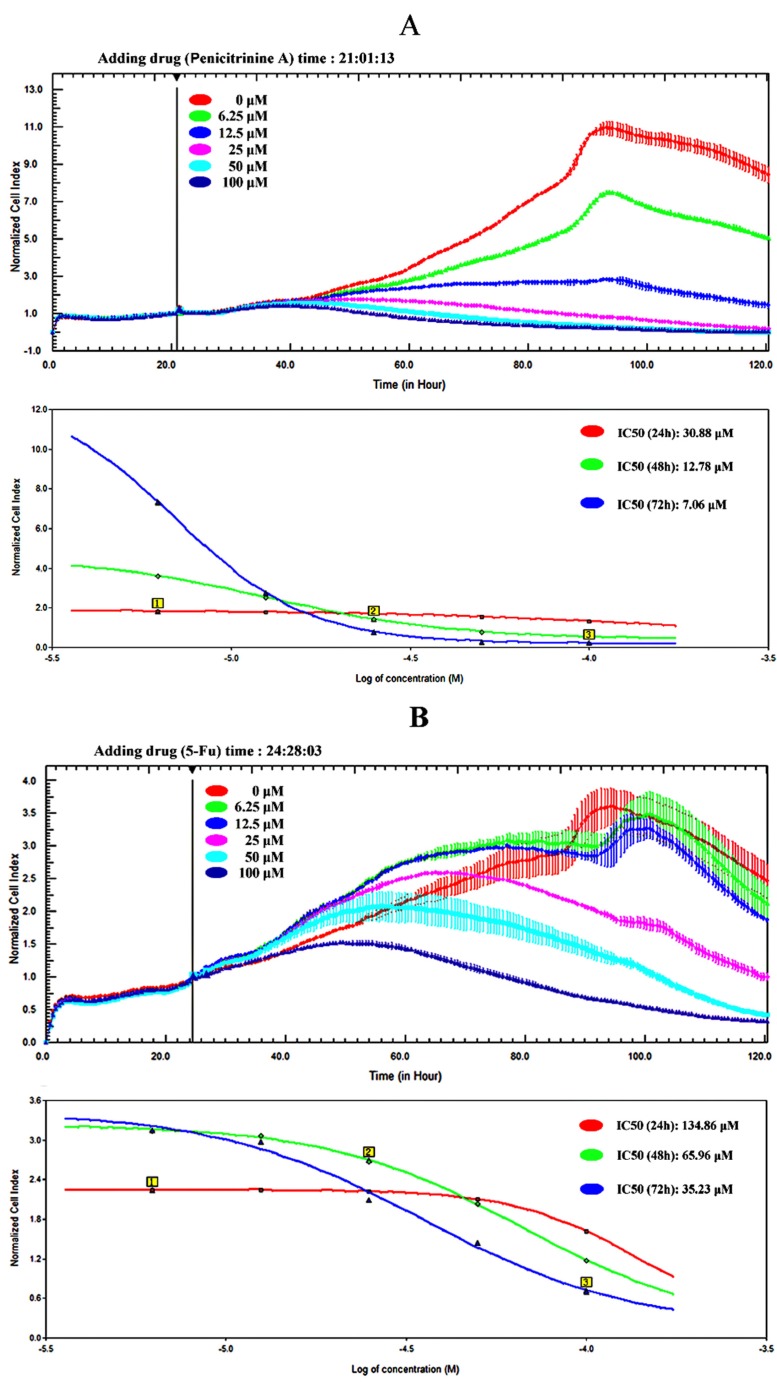
The viability of A-375 cells treated with penicitrinine A (**A**) or 5-Fu (**B**) was determined by RTCA assay.

In order to accurately characterize anti-proliferative potential of penicitrinine A in A-375 cells, we developed an RTCA assay to determine alive cell number. The effect of the first-line chemotherapy drug 5-Fu was also tested as a positive control on A-375 cells. The results showed that the IC_50_ of A-375 with penicitrinine A treatment for 24 h, 48h and 72 h was 30.88 μM, 12.78 μM and 7.06 μM, respectively, while the IC_50_ with 5-Fu treatment for 24 h, 48h and 72 h was 134.86 μM, 65.96 μM and 35.23 μM, respectively (Figure 5), revealing that penicitrinine A inhibits A-375 cells growth in a dose- and time-dependent manner and has more potent anticancer activity than 5-Fu.

### 2.5. Penicitrinine A Induces Significant Apoptotic Morphological Changes in A-375 Cells

**Figure 6:**
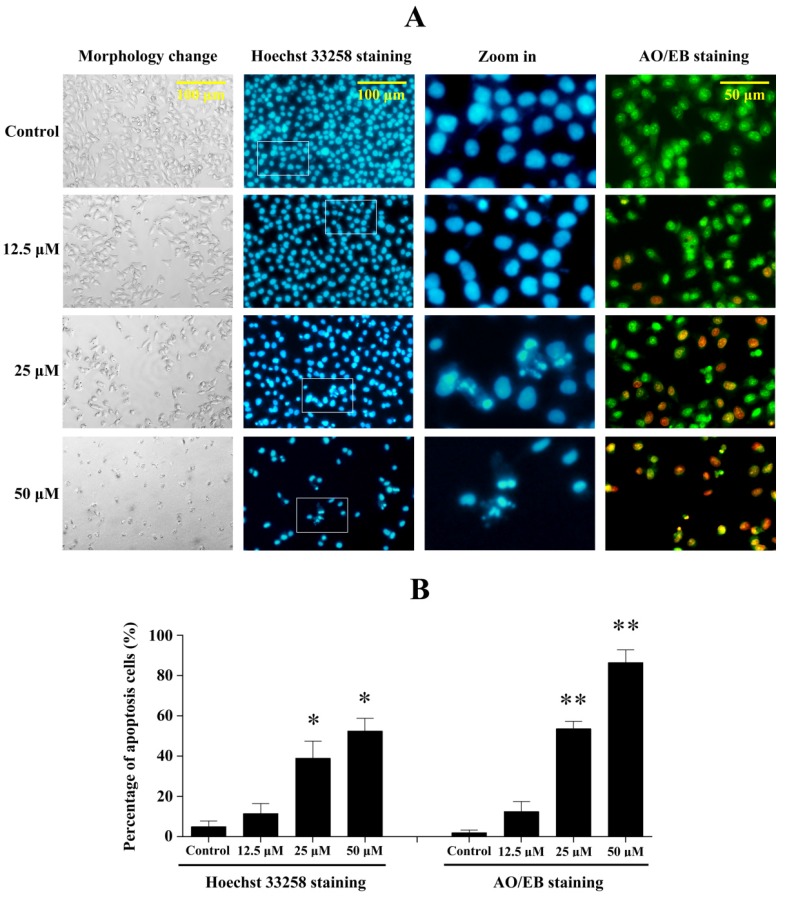
Penicitrinine A induced significant apoptotic morphological changes. (**A**) After exposed to 12.5, 25 and 50 μM penicitrinine A for 24 h, A-375 cells were stained by Hoeschest 33258 or AO/EB. Photos were taken under an inverted fluorescence microscope; (**B**) Quantification of the proportion of apoptotic cells detected by Hoechst 33258 staining and AO/EB staining. The values (means ± SD, *n* = 3) differed significantly (* *p* < 0.05; ** *p* < 0.01). The third column in Figure A was amplified from the second column for ten fold.

To determine whether the growth inhibitory activity of penicitrinine A was related to the induction of apoptosis, we further examined the changes in cell morphology after exposure to penicitrinine A. As shown in Figure 6, 24 h after exposure to 5 μM, 10 μM and 20 μM of penicitrinine A, A-375 cells began to show cell shrinkage, rounding and fragmentation, thus taking on the typical appearance of apoptotic cells when compared to untreated cells. We also analyzed cell morphology changes by Hoechst 33258 and AO/EB staining, and the results showed penicitrinine A-treated cells exhibited chromatin condensation and nuclear fragmentation, which were indicative of apoptosis.

### 2.6. Penicitrinine A Induces Apoptosis in A-375 Cells

**Figure 7:**
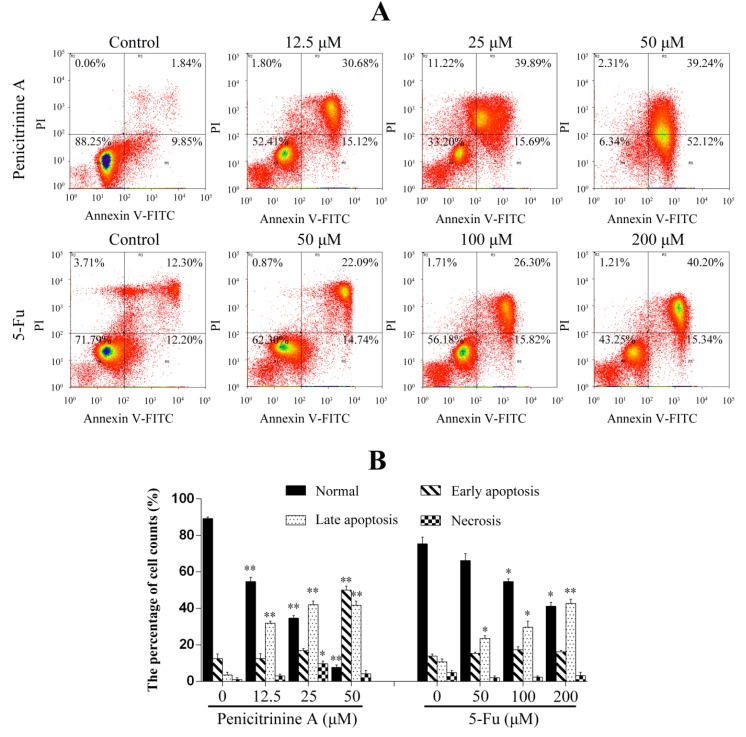
Penicitrinine A induced A-375 cells apoptosis. (**A**) A-375 cells were treated with penicitrinine A (12.5 μM, 25 μM and 50 μM) for 24 h, stained by annexin-V/PI and analyzed by flow cytometry, 5-Fu (50 μM, 100 μM and 200 μM) as a positive control; (**B**) Densitometry of cell counts. The values (means ± SD, *n* = 3) differed significantly (* *p* < 0.05; ** *p* < 0.01).

In order to determine the apoptotic cell death in A-375 cells induced by penicitrinine A, Annexin-V/PI double staining was performed. The results showed that apoptosis rates of A-375 cells changed from 45.80%, 55.58% to 91.36% when treated with 12.5 μM, 25 μM and 50 μM of penicitrinine A (Figure 7). However, the apoptosis rates only changed from 24.50%, 36.83% to 55.54% when treated with 50 μM, 100 μM and 200 μM of 5-Fu (Figure 7). The above results demonstrated that penicitrinine A could induce apoptosis much more effectively than 5-Fu in a dose-dependent manner.

### 2.7. Penicitrinine A Modulates Apoptosis -Related mRNA and Protein in A-375 Cells

**Figure 8:**
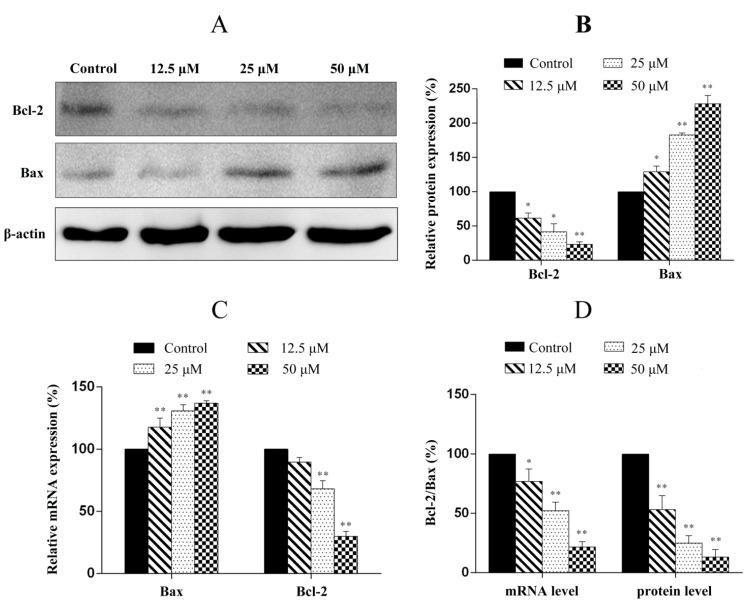
Western blot and RT-qPCR analysis of the apoptosis-associated molecules in A-375 cells. (**A**) After cells were treated with different concentrations of penicitrinine A for 24 h, western blot analysis was performed using antibodies against Bcl-2, Bax and β-actin; (**B**) Densitometric analysis of Bcl-2 and Bax at protein levels; (**C**) A-375 cells were treated with 0, 12.5, 25 and 50 µM penicitrinine A for 24 h. The mRNA levels from whole cell lysates were analyzed by RT-qPCR, GAPDH was used as a loading control; (**D**) Summary of the ratio of Bcl-2 to Bax as demonstrated by histograms. Data are expressed as mean ± SEM (*n* = 3). * *p* < 0.05, ** *p* < 0.01 *vs.* the control group.

We next examined the protein and mRNA levels of genes related to the apoptosis such as Bcl-2 and Bax using Western blot and real-time quantitative PCR (RT-qPCR). As shown in Figure 8, the anti-apoptotic gene Bcl-2 was down-regulated and the pro-apoptotic gene Bax was up-regulated at both protein and mRNA levels after the cells were treated with penicitrinine A (Figure 8A–C). The ratio of Bcl-2/Bax, which is a key factor regulating apoptosis, was decreased with the increased concentration of penicitrinine A (Figure 8D). These findings suggested that penicitrinine A-induced apoptosis might be involved in the mitochondrion-mediated apoptosis pathway.

### 2.8. Penicitrinine A Inhibits the Cell Migration and Invasion in A-375 Cells

**Figure 9:**
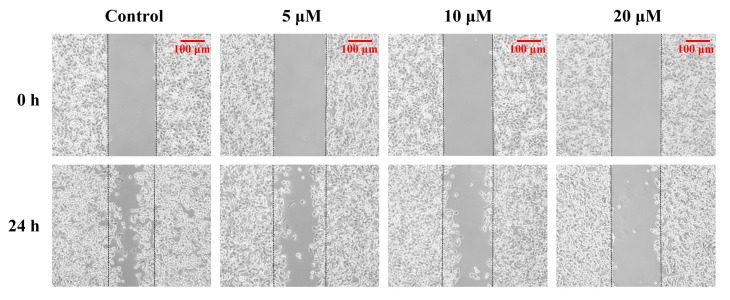
Effects of penicitrinine A on the wound healing migration of A-375 cells. A wound was introduced by scraping confluent cell layers with a pipet tip. A-375 cells were incubated with penicitrinine A (5, 10 or 20 μM) for 24 h. Photos of the wounds were taken at 0 and 24 h under an inverted microscope.

Since malignant melanomas are characterized by frequent metastasis, we further determined the function of penicitrinine A in cell migration and invasion. We first performed a wound healing assay in A-375 cells and observed that the cells treated with penicitrinine A were significantly inhibited to migrate to the wounded area in a dose-dependent manner (Figure 9).

**Figure 10:**
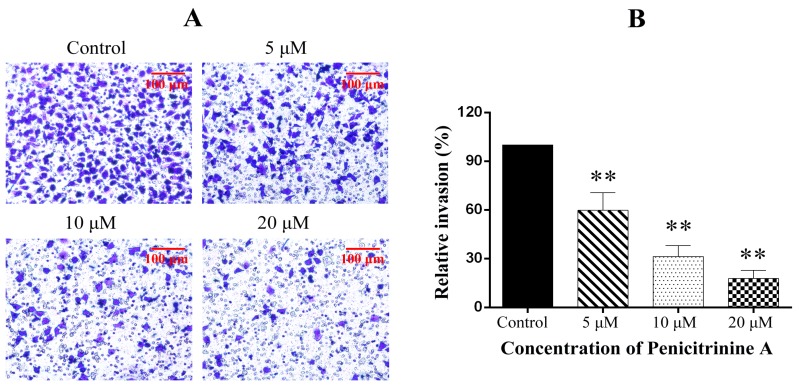
Effect of penicitrinine A on the transwell invasion assay of A-375 cells. (**A**) A-375 cells incubated with penicitrinine A (5, 10, or 20 μM) were plated in the upper chambers of Matrigel-coated transwell insert for 24 h. The lower chamber contained 20% FBS as a chemoattractant in the medium. After incubated for 24 h, cells were fixed by methanol, stained with crystal violet, and observed under microscope; (**B**) The invaded A-375 cells were counted in five random fields in each treatment, and data were calculated from three independent experiments. Data are presented as mean ± SD of three independent experiments. * *p* < 0.05, ** *p* < 0.01 compared with the untreated control.

We further performed the trans-well assay to evaluate the inhibitory effect of penicitrinine A on the invasive features of A-375 cells. The results showed that penicitrinine A suppressed the invasion of A-375 cells across the Matrigel-coated filter in a dose-dependent manner. As shown in Figure 10, the cell invasion was reduced by 40.2%, 69.3% and 81.6% when treated with penicitrinine A at doses of 5, 10 and 20 μM, compared with the untreated control.

### 2.9. Penicitrinine A Modulates Metastatic-Related Proteins in A-375 Cells

**Figure 11:**
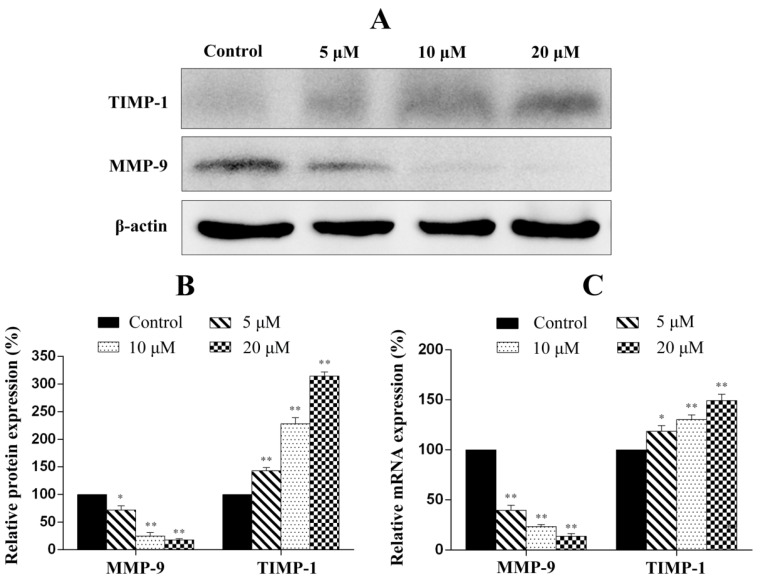
RT-qPCR and western blot analysis of the metastatic-associated molecules in A-375 cells. (**A**) After cells were treated with different concentration of penicitrinine A for 24 h, western blot analysis was performed using antibodies against TIMP-1, MMP-9 and β-actin; (**B**) Densitometric analysis of MMP-9 and TIMP-1 at protein levels; (**C**) A-375 cells were treated with 0, 5, 10 and 20 µM penicitrinine A for 24 h. The mRNA level from whole cell lysates was analyzed by RT-qPCR, GAPDH was used as a loading control. Data are expressed as mean ± SEM (*n* = 3). * *p* < 0.05, ** *p* < 0.01 *vs.* the control group.

To evaluate whether penicitrinine A is able to suppress the metastatic potential of highly metastatic cancer cells, the modulation of the protein and mRNA expression levels of genes associated with metastasis was examined using western blot analysis and RT-qPCR. The results showed that penicitrinine A treatment leads to the down-regulation of MMP-9 in both protein the mRNA levels, whereas the expression of TIMP-1 was up-regulated in a dose-independent manner, demonstrating that penicitrinine A suppresses cell migration by up-regulating TIMP-1 and down-regulation MMP-9 levels in human melanoma A-375 cell line, as shown in Figure 11.

### 2.10. Discussion

In recent years, the use of marine natural products for cancer prevention and therapy has received a great deal of attention owing to their various health benefits and noticeable lack of toxicity and side effects [23]. Penicitrinine A, a novel alkaloid with a unique spiro skeleton, was isolated and purified from marine-derived fungus *P. citrinum*. Further molecular mechanism study revealed that penicitrinine A could induce apoptosis of multiple human tumor types, especially melanoma cancer cell line A-375, under the regulation of Bcl-2 family, and inhibit the metastasis of A-375 through up-regulation of TIMP-1 and down-regulation of MMP-9, suggesting that penicitrinine A might be a potential antitumor agent.

The structure of penicitrinine A was established mainly by NMR and HRESIMS analyses. Moreover, we presumed a plausible biosynthetic pathway of penicitrinine A by Diels-Alder [] reaction. Until now, penicitrinine A is the first metabolite biosynthetized from citrinin and tetramic acid derivatives in nature.

Cytotoxic activity is regarded as the first indicator in identifying anticancer drugs [24]. In the previous studies, we evaluated the cytotoxic activities of the metabolites obtained from *P. citrinum* [19,21,22,25]. Among them, dicitrinone B showed potent anticancer efficacy against multiple human tumor types. Considering penicitrinine A has a similar structural part to dicitrinone B, we, for the first time, detected the effects of penicitrinine A on the growth against human stomach, lung, colon, melanoma, oral epidermoid, liver, nasopharynx, esophagus, breast and lymphoma cancer cell lines. Similar to the results of dicitrinone B, different tumor cell lines had different levels of proliferation inhibition after being treated with penicitrinine A. Most interestingly, the most sensitive cell line was also melanoma A-375 cells. We further studied the anticancer activity of penicitrinine A and the first-line chemotherapy drug 5-Fu on A-375 cells with RTCA assay, the IC_50_ value for 48 h under penicitrinine A (12.78 μM) treatment presented slightly lower than dicitrinone B (13.38 μM) and much less than 5-Fu (65.96 μM), suggesting that penicitrinine A has stronger cytotoxic activity than dicitrinone B.

Apoptosis is a highly conserved process that plays an important role in the regulation of the cellular activities of eukaryotes and is characterized by chromatin condensation [26,27]. Several studies reported that apoptosis in the A-375 cells presented typical apoptotic morphological changes with cell shrinkage, nuclei blebbing, chromatin condensation, and nuclear fragmentation [28,29]. Our data indicated penicitrinine A induced A-375 cells apoptosis, since cells changed nuclear morphology by chromatin condensation using Hoechst 33258 and AO/EB staining. Annexin V-FITC/PI staining results further confirmed that treatment with penicitrinine A has more remarkable effect in increasing the early and late apoptotic rates than 5-Fu. Apoptotic processes can be divided into two major pathways: the extrinsic pathway and the intrinsic pathway [30,31]. The intrinsic pathway is triggered by a large number of intracellular signals located in the endoplasmic reticulum or mitochondria [32]. One of the most important regulators of the mitochondrion-mediated pathway is the Bcl-2 protein family [33]. Members of this family can either possess pro-apoptotic characteristics, such as Bax, Bak, Bad and Bid, or possess anti-apoptotic characteristics, such as Bcl-2, Bcl-XL, Bcl-W and Mcl-1. Typically, the ratio of Bcl-2 and Bax indicates the threshold sensitivity of cells to the induction of apoptosis via the intrinsic pathway [34]. Our data clearly showed that penicitrinine A treatment resulted in a dose-dependent increase in the level of Bax with a concomitant decrease in Bcl-2 level and finally the decrease of Bcl-2/Bax ratio, demonstrating that penicitrinine A could promote the apoptosis process by changing the expression ratio of Bcl-2/Bax, but the details of the mechanisms need further study.

Malignant melanomas are characterized by a high capacity for metastasis [35]. In the present study, we observed that penicitrinine A could effectively inhibit the potential invasion and migration of A-375 cells by wound healing assay and trans-well assay. Degradation of the extracellular matrix is thought to be a key mechanism in tumor metastasis, and the process could be regulated by the expression of MMPs protein families and their specific inhibitor TIMPs [36]. Our results identified that penicitrinine A treatment significantly suppressed the expression of MMP-9 but promoted the expression of its inhibitor TIMP-1, at both transcriptional and translational levels in A-375 cells. Moreover, the increase of metastatic melanoma cells was reported to be associated with enhanced tendency to undergo epithelial-mesenchymal transition (EMT) [37,38,39]. In fact, we also analyzed several EMT biomarkers such as E-cadherin, N-cadherin and Snail1 at transcriptional level, and found penicitrinine A could affect the EMT process by reducing the expression of N-cadherin and Snail1 (data not shown). However, the detail mechanism needs further study.

In conclusion, this is the first report that clearly described the structure and the antitumor properties of penicitrinine A, as well as identified its mechanism in a tumor model. Penicitrinine A not only induced cell apoptosis by regulated Bcl-2 and Bax secretion but inhibited cell metastasis through suppressed MMP-9 activity, suggesting that penicitrinine A is a promising chemotherapeutic agent to the treatment of melanoma A-375 cells.

## 3. Experimental Section

### 3.1. Isolation of Penicitrinine A

#### 3.1.1. General Experimental Procedures

Optical rotations were obtained from a Shenguang SGW-1 digital polarimeter (Shenguang, Shanghai, China). UV spectra were recorded on a Shimadzu UV-2450 spectrophotometer (Shimadzu, Kyoto, Japan). IR spectra were recorded on a Nicolet Avatar 670 spectrophotometer (Thermo Fisher, Waltham, MA, USA). ^1^H-NMR, ^13^C-NMR, DEPT spectra and 2D-NMR were recorded on a BRUKER BIOSPIN AVANCE III spectrometer (Bruker, Karlsruhe, Germany). Using TMS as the internal standard. HRESIMS were obtained by an AGILENT 1200/Q-TOF 6510 LC mass spectrometer (Agilent, Palo Alto, CA, USA). Semipreparative HPLC was performed using an ODS column (ODS-A, 10 × 250 mm, 5 µm) (YMC, Tokyo, Japan) at 5 mL/min.

#### 3.1.2. Fungal Material

The fungus *P. citrinum* was isolated from marine sediments collected from Langqi Island, Fujian, China. It was identified according to its morphological characteristics and ITS by Beijing Sunbiotech Co. Ltd (Beijing, China), and preserved in our laboratory at −80 °C. The producing strain was prepared on Martin medium and stored at 4 °C.

#### 3.1.3. Fermentation and Extraction

The fungus was cultured under static conditions at 28 °C for 30 days in 1000-mL conical flasks containing the liquid medium (400 mL/flask), composed of glucose (10 g/L), maltose (20 g/L), mannitol (20 g/L), monosodium glutamate (10 g/L), KH_2_PO_4_ (0.5 g/L), MgSO_4_·7H_2_O (0.3 g/L), yeast extract (3 g/L), and seawater. The fermented whole broth (60 L) was filtered through cheesecloth to separate supernatant from mycelia. The former was extracted two times with EtOAc to give an EtOAc solution that was concentrated under reduced pressure to give a crude extract (32.0 g).

#### 3.1.4. Purification of Penicitrinine A

The broth extract (32.0 g) was separated into 11 fractions on a Si gel column using a step gradient elution of petroleum ether, CH_2_Cl_2_, and MeOH. Fraction 7 (4.2 g) eluted with CH_2_Cl_2_:MeOH = 100:1 was further purified on a Si gel column using a step gradient elution. Subfraction 7–6 (400 mg) was purified by semipreparative HPLC (75% MeCN containing 0.1% TFA) to yield compound **1** (97.9 mg).

#### 3.1.5. Spectral Data

*Penicitrinine A*: yellow oil (CHCl_3_); ${\left[\alpha \right]}_{D}^{20}$ +74.0° (*c* 0.20, CHCl_3_); UV (MeOH) λ_max_ (log ɛ) 252 (2.81), 287 (2.94) nm; IR (KBr) ν_max_ 3440, 2970, 2929, 2856, 1724, 1659, 1601, 1462, 1377, 1343, 1123, 1046 cm^−1^; ^1^H- and ^13^C-NMR data (see Table 1); HRESIMS *m*/*z* 484.2711 [M − H]^−^ (calcd. for C_28_H_38_NO_6_, 484.2705).

### 3.2. Cell Culture and Treatment

Malignant melanoma A-375 cells were obtained from Shanghai Cell Resource Center (Shanghai, China) and were cultured in DMEM (Hyclone, Thermo Scientific, Beijing, China) medium. The medium was supplemented with 10% fetal bovine serum (Hyclone) and the cells were incubated in a humidifier with 5% CO_2_ at 37 °C. At various concentrations after the treatment, the cells were processed for the analyses of cell cytotoxicity, morphology changes, apoptosis and motility.

### 3.3. WST-1 Cell Proliferation Assay

Cytotoxic activity was evaluated by the WST-1 cell proliferation assay kit (Roche, Indianapolis, IN, USA). Briefly, cells were cultured in 96-well plate, and treated with gradient concentrations of penicitrinine A. After 48 h, one-tenth volume WST-1 solution was added. After cells were incubated at 37 °C for4 h, the absorbance at 450 nm was measured.

### 3.4. RTCA Cytotoxicity Assay

Cell proliferation was performed using the DP version of the xCELLigence real time cell analyzer RTCA (ACEA, San Diego, CA, USA), which records changes in impedance (reported as a cell index-CI) over a prolonged time course in a noninvasive system. Briefly, the background impedance of RTCA DP E-Plates 16 (ACEA, SanDiego, CA, USA) was performed using the standard protocol provided in the software with 50 μL of media. 5 × 10^3^ A-375 cells were seeded with 100 μL of DMEM and left to equilibrate at room temperature for 30 min. Cells were allowed to grow for 24 h before adding penicitrinine A or 5-Fu to the cultures at the indicated concentrations in duplicate. The CI of the proliferating cells was recorded and expressed as mean of CI normalized to the CI recorded at the time of A-375 cells treatment compared to untreated cells.

### 3.5. Morphological Analysis

#### 3.5.1. Hoechst 33258 Staining

Hoechst labeling of cells was used to detect apoptotic nuclei by evaluation of nuclear morphology. Cells (1 × 10^5^ cells/well) were plated in six-well plates then treated with gradient concentrations of penicitrinine A for 24 h. After incubation, the medium was removed and cells were fixed in a solution of methanol and acetic acid with 3:1 for 10 min at room temperature. To assess specific apoptosis, Hoechst 33258 (10 μg/mL) (Sigma, St. Louis, MO, USA) was added to each well and further incubated at room temperature for 15 min in the dark. After washed with PBS twice, the cells were observed under an Olympus fluorescence microscope (Olympus America Inc., Center Valley, PA, USA).

#### 3.5.2. AO/EB Staining

A-375 cells were seeded in six-well plates with different concentrations of penicitrinine A (12.5, 25 and 50 μM) at 1 × 10^5^ cells/well and incubated at 37 °C under 5% atmospheric CO_2_ concentration for 24 h. AO/EB solution was prepared at 100 μg/mL of each reagent. Cells were harvested and stained with 100 μL of AO/EB solution for 15 min, then washed twice with PBS and observed under an Olympus fluorescence microscope (Olympus America Inc., Center Valley, PA, USA). Green color indicated live cells, whereas cells with orange and red color were apoptotic and necrotic cells, respectively.

### 3.6. Flow Cytometry Analysis

Apoptosis in A-375 cells was measured by using the Annexin V FLUOS staining Kit (Roche, Indianapolis, IN, USA). Briefly, cells were exposed to different concentrations of penicitrinine A in six-well plates, were washed twice with PBS solution and then resuspended at a concentration of 1 × 10^6^ cells/mL in binding buffer. Subsequently, 1000 μL of the cell suspension was mixed with 20 μL of Annexin V-Flous and 20 μL of PI. After incubated at room temperature for 15 min, apoptotic cells were determined using a FACScan (Beckman Coulter, Brea, CA, USA).

### 3.7. Wound Healing Assay

To assess the motility of cells, a wound healing assay was performed [40]. A-375 cells (1 × 10^6^ cells/mL) were seeded in six-well plates. After 24 h, the center of the cell monolayers was scraped with a sterile micropipette tip to create a straight gap of constant width. Then, each well was washed with PBS, and A-375 cells were exposed to various concentrations of penicitrinine A (5, 10 or 20 μM). Wound closure was photographed at 0 h and 24 h with an inverted microscope (Nikon, Tokyo, Japan).

### 3.8. Trans-Well Assay

Tumor cell invasion were performed in trans-well chambers (Millipore, Billerica, MA, USA) according to the method reported previously [41], with some modifications. The upper trans-well chambers were coated with Matrigel (BD Biosciences, Franklin Lakes, NJ, USA), A-375 cells (6 × 10^5^ cells/well) and 5, 10 or 20 μM of penicitrinine A were suspended in DMEM (100 μL, serum free), placed in the chambers, and the medium of the lower plates also contained 20% fetal bovine serum as a chemoattractant incubated. The invaded cells on the lower chamber were fixed with methanol and stained with 5% crystal violet. Then, the cells on the upper surface of the filter were completely wiped away with a cotton swab and photographed under a microscope (Nikon, Tokyo, Japan).

### 3.9. Real-Time Quantitative PCR

For RT-qPCR, total RNA from control as well as cells treated with gradient concentrations of penicitrinine A was extracted using TRIzol reagent (Life Technologies, Carlsbad, CA, USA), according to the manufacturer’s protocol. cDNAs were synthesized using the PrimeScript™ RT reagent kit (Takara, Otsu, Shiga, Japan). Quantitative PCR was performed using the ABI Step one plus System with miScript SYBR Green PCR Kit (QIAGEN, Hilden, Germany) and primers were listed in Table 3.

**Table 3:** Primers for the genes used in RT-qPCR test.

Gene		Sequences
Bcl-2	forward	5′-CGACTTTGCAGAGATGTCCA-3′
	reverse	5′-ATGCCGGTTCAGGTACTCAG-3′
Bax	forward	5′-CCTTTTCTACTTTGCCAGCAAAC-3′
	reverse	5′-GAGGCCGTCCCAACCAC-3′
MMP-9	forward	5′-CCTGGAGACCTGAGAAC-3′
	reverse	5′-CAGGGACAGTTGCTTCT-3′
TIMP-1	forward	5′-ACTCTTGCACATCACTACCT-3′
	reverse	5′-AAACACTGTGCATTCCTC-3′
GAPDH	forward	5′-GAAGGTGAAGGTCGGAGTC-3′
	reverse	5′-GAAGATGGTGATGGGATTTC-3′

The mRNA expression levels were calculated by the 2^−ΔΔCT^ method and expressed in relative quantification units. Each reaction was amplified in triplicate and relative mRNA levels were normalized to those of GAPDH.

### 3.10. Western Blot

A-375 cells were plated in six-well plates at a density of 1 × 10^6^ cells per well and incubated with various concentrations of penicitrinine A for 24 h. The cells were collected, lysed in RIPA buffer. Total proteins were separated by 12% SDS-PAGE and then transferred to the NC membrane. The membranes were blocked with 5% (w/v) nonfat dry milk in PBST for one hour and changed to an appropriate dilution of specific primary antibodies against Bcl-2 (1:1000, cell signaling technology), Bax (1:1000, cell signaling technology), MMP-9 (1:1000, cell signaling technology), TIMP-1 (1:1000, cell signaling technology) and β-actin (1:1000, cell signaling technology) in milk overnight at 4 °C. The secondary antibodies were peroxidase-conjugated anti-rabbit IgG (1:5000). Signals were detected by an electrochemiluminescence (ECL) reagent using Image Station 4000MM digital darkroom system (Kodak, St. Rochester, NY, USA).

### 3.11. Statistical Analysis

The values were presented as means ± SD. Statistical analyses were performed using student’s *t*-test. *p* < 0.05 was considered statistically significant for all tests.

## 4. Conclusions

This study investigated a new compound penicitrinine A isolated and identified from marine fungus *P. citrinum*. We found that penicitrinine A has cytotoxic activity on a wide range of tumor cells, including A-375 cells. Further studies have shown that penicitrinine A induced A-375 cells apoptosis by regulating the Bcl-2 family proteins, and significantly suppressed the migration of A-375 cells through regulating the expression of MMP-9 and its specific inhibitor TIMP-1. These findings demonstrated the biological activity of penicitrinine A in *in vitro* models of cancer apoptosis and metastasis, and showed that penicitrinine A, which has a unique chemical structure, could serve as a lead compound for rational drug design and for future development of anticancer agents.

## Abbreviations

HPLC: high performance liquid chromatography
NMR: nuclear magnetic resonance
HRESIMS: high resolution electrospray ionization mass spectroscopy
DEPT: distortionless enhancement by polarization transfer
HMBC: heteronuclear multiple bond connectivity
HMQC: heteronuclear multiple quantum connectivity
5-Fu: 5-fluorouracil
AO/EB: acridine orange/ethidium bromide
MMP-9: matrix metallopeptidase 9
TIMP-1: metallopeptidase inhibitor 1
RTCA: real time cell analyzer
CTLA-4: cytotoxic T-lymphocyte-associated protein 4
PD-1: programmed cell death 1
COSY: (homonuclear chemical shift) correlation spectroscopy
NOESY: nuclear overhauser enhancement spectroscopy
GAPDH: glyceraldehyde-3-phosphate dehydrogenase
FBS: fetal bovine serum
PI: propidium iodide
TMS: tetramethylsilane
DMEM: dulbecco’s modified eagle’s medium
PBS: phosphate buffered solution
